# Acteoside alleviates slow transit constipation with attenuation of NLRP3 inflammasome activation and preservation of enteric neurons

**DOI:** 10.3389/fphar.2026.1921808

**Published:** 2026-09-07

**Authors:** Baohong Zhao, Yaqian Ping, Xiaohui Dou, Ziying Jiang, Fangxu Yin, Daqing Sun

**Affiliations:** 1 Department of Pediatric Surgery, Tianjin Medical University General Hospital, Tianjin, China; 2 Department of General Surgery, Yuhuangding Hospital, Yantai, China

**Keywords:** acteoside, enteric nervous system, inflammatory response, NLRP3 inflammasome, slow transit constipation

## Abstract

**Background:**

Slow transit constipation (STC) is characterized by delayed intestinal transit and impaired defecation, with enteric nervous system (ENS) injury and inflammatory dysregulation contributing to its pathogenesis. Acteoside (ACT), a natural phenylethanoid glycoside, has anti-inflammatory and neuroprotective properties. However, its therapeutic effect and mechanism in STC remain unclear.

**Methods:**

The effects of ACT were investigated in a loperamide (LOP)-induced STC-like mouse model. Intestinal motility, colonic morphology, and ENS integrity were evaluated by functional assays, hematoxylin and eosin (H&E) staining, and immunofluorescence. Transcriptomic analysis was performed to screen ACT-responsive inflammatory pathways. NOD-like receptor family pyrin domain-containing 3 (NLRP3) inflammasome activation and cytokine production were assessed by Western blotting, enzyme-linked immunosorbent assay (ELISA), and quantitative reverse transcription polymerase chain reaction (qRT-PCR). *Nlrp3*-knockout (*Nlrp3*-KO) mice were used for genetic validation.

**Results:**

ACT shortened defecation latency, increased fecal water content and pellet number, and improved intestinal propulsion. ACT also alleviated colonic histopathological injury, restored muscular layer thickness, preserved HuC/D-positive myenteric neurons, and increased PGP9.5-positive nerve fiber density. Transcriptomic analysis identified NLRP3-related inflammatory signaling within broader high-dose ACT-responsive transcriptional changes. At the molecular level, high-dose ACT reduced the protein levels of NLRP3, cleaved-caspase-1, and cleaved-interleukin-1β (IL-1β) expression and reduced IL-1β, interleukin-6 (IL-6), and tumor necrosis factor-α (TNF-α) production. *Nlrp3* deficiency partially reproduced ACT-mediated protection against STC-like phenotypes, inflammasome activation, and ENS injury, whereas ACT retained additional protective effects in *Nlrp3*-KO mice, suggesting that NLRP3-independent mechanisms may also contribute.

**Conclusion:**

ACT alleviates LOP-induced STC-like injury, and this protection is associated with attenuation of NLRP3 inflammasome activation and preservation of ENS integrity, while additional NLRP3-independent mechanisms may also contribute.

## 1 Introduction

Slow transit constipation (STC) is a major subtype of chronic constipation characterized by delayed intestinal transit, infrequent defecation, hard stools, and difficult bowel evacuation (Vlismas et al., 2024; Bharucha and Lacy, 2020; Syllaios et al., 2026). Current therapeutic approaches mainly include dietary modification, osmotic or stimulant laxatives, intestinal secretagogues, prokinetic agents, and surgery in selected refractory cases (Syllaios et al., 2026; Vlismas et al., 2024; Bharucha and Lacy, 2020; Chang et al., 2023). Although these strategies are clinically useful, treatment responses remain heterogeneous, and long-term management is often limited by insufficient efficacy, symptom recurrence, poor adherence, or adverse effects (Syllaios et al., 2026; Bharucha and Lacy, 2020; Chang et al., 2023). In this context, bioactive compounds derived from natural products, including medicine food homologous resources, have attracted increasing attention as potential interventions for functional constipation (Yu et al., 2025). Therefore, the development of safer and more effective therapeutic strategies remains an important focus in STC research.

The enteric nervous system (ENS), the intrinsic neural network of the gastrointestinal tract, plays a central role in coordinating intestinal motility, secretion, and local neuroimmune homeostasis (Sharkey and Mawe, 2023; Wang et al., 2022; Jacobson et al., 2021). In STC, delayed intestinal transit is closely associated with enteric neuromuscular abnormalities. Previous pathological studies have reported alterations in enteric nerves, myenteric plexuses, and interstitial cells of Cajal in patients with STC, supporting the view that STC is not merely a disorder of stool retention but is closely linked to enteric neuropathy and neuromuscular remodeling (Wedel et al., 2002; He et al., 2000). Therefore, preserving myenteric neurons and enteric nerve fibers may represent a disease-relevant strategy for improving intestinal motility.

Inflammatory signaling is increasingly recognized as an important contributor to gastrointestinal neuromuscular dysfunction. Enteric neurons are anatomically and functionally embedded within a neuroimmune microenvironment, in which immune-derived cytokines can modulate neuronal excitability, neurotransmission, and intestinal contractility (Xia et al., 1999; Wang et al., 2022; Jacobson et al., 2021). The NOD-like receptor family pyrin domain-containing 3 (NLRP3) inflammasome is a key innate immune platform that promotes caspase-1 activation and the subsequent maturation of interleukin-1β (IL-1β) and IL-18 (Xu and Núñez, 2023; He et al., 2016; Kelley et al., 2019). Dysregulated NLRP3 activation has been implicated in intestinal inflammation, barrier dysfunction, and inflammatory tissue injury (Zhen and Zhang, 2019; Li et al., 2025). Given that IL-1β, IL-6, and TNF-α can directly affect enteric neuronal activity and gastrointestinal motility, NLRP3 inflammasome activation may represent an important inflammatory mechanism linking ENS injury to delayed intestinal transit (Xia et al., 1999; Akiho et al., 2011; Tjwa et al., 2003).

Acteoside (ACT), also known as verbascoside, is a natural phenylethanoid glycoside with antioxidant, anti-inflammatory, immunomodulatory, and neuroprotective properties (Mohammed et al., 2025; Xiao et al., 2022). In experimental intestinal inflammation, ACT has been shown to alleviate dextran sulfate sodium-induced colitis and suppress inflammatory signaling (Guo et al., 2022). Notably, studies using central nervous system injury models have reported that ACT or verbascoside attenuates neuroinflammation by inhibiting microglial HMGB1/TLR4/NLRP3 signaling in ischemic stroke, suppressing NLRP3 activation after intracerebral hemorrhage, and blocking NF-κB-p65-mediated neuroinflammation in Alzheimer’s disease models (Liao et al., 2024; Zhou et al., 2021; Chen et al., 2022). These findings suggest that ACT may be particularly relevant to inflammation-associated neuronal injury. However, whether ACT ameliorates STC by protecting enteric neurons, and whether inhibition of the NLRP3 inflammasome is involved in this process, remain unclear.

Based on these observations, we hypothesized that ACT could alleviate loperamide-induced STC-like phenotypes by suppressing NLRP3 inflammasome activation, thereby reducing inflammatory cytokine production and preserving the integrity of myenteric neurons and enteric nerve fibers. To test this hypothesis, we evaluated the effects of ACT on defecation parameters, intestinal propulsion, colonic histopathology, and ENS integrity in a loperamide-induced STC-like mouse model. Transcriptomic analysis was performed to identify potential inflammatory targets, followed by validation of NLRP3 inflammasome activation and cytokine production. Furthermore, *Nlrp3*-knockout (*Nlrp3*-KO) mice were used to determine whether NLRP3 signaling contributes to ACT-mediated improvement of STC-like phenotypes and myenteric neuroprotection.

## 2 Materials and methods

### 2.1 Materials and reagents

ACT (purity ≥98%, Cat# CFN97048) was purchased from ChemFaces (Wuhan, China). LOP (Cat# L129465) was obtained from Aladdin Biochemical Technology Co., Ltd. and used to establish the LOP-induced STC-like mouse model. Primary antibodies used for Western blotting included anti-NLRP3 (D4D8T, 1:1000, Cell Signaling Technology), anti-cleaved-caspase-1 (Cat# P79884R2, 1:1000, Abmart), anti-IL-1β (Cat# PC25615, 1:1000, Abmart), anti-cleaved-IL-1β (Cat# P5020-1R1, 1:1000, Abmart), and anti-β-actin (Cat# 20536-1-AP, 1:10000, Proteintech). The horseradish peroxidase (HRP)-conjugated secondary antibody used for Western blotting was purchased from Beyotime (Cat# A0208, 1:10000, China). Primary antibodies used for immunofluorescence staining included anti-HuC/D (Cat# 24992-1-AP, 1:200, Proteintech) for labeling myenteric neurons and anti-PGP9.5 (Cat# 14730-1-AP, 1:200, Proteintech) for detecting enteric nerve fibers. An antifade mounting medium containing 4′,6-diamidino-2-phenylindole (DAPI) (Cat# S2110, Solarbio) was used for nuclear counterstaining. The fluorescent secondary antibody used for immunofluorescence detection was CoraLite 488-conjugated goat anti-rabbit IgG (H + L) (Cat# SA00013-2, 1:100, Proteintech). Enzyme-linked immunosorbent assay (ELISA) kits for IL-1β (Cat# EM30300S), IL-6 (Cat# EM30325S), and TNF-α (Cat# EM30536S) were purchased from Shanghai Weiao Biotechnology Co., Ltd (Shanghai, China). For the intestinal propulsion test, India ink (Cat# S30881, Shanghai Yuanye Bio-Technology Co., Ltd., Shanghai, China) was diluted in sterile double-distilled water (ddH_2_O) to prepare a fresh working suspension before oral gavage. All chemical reagents were of analytical grade or higher, and sterile ddH_2_O was used throughout the experiments.

### 2.2 Animals and experimental design

Male C57BL/6 mice aged 6–8 weeks were purchased from Sikebeisi Biotechnology Co., Ltd (Henan, China). *Nlrp3*-KO mice on the same genetic background were obtained from Shanghai Model Organisms Center, Inc (Shanghai, China), and knockout validation was provided by the supplier. All mice were housed in a specific pathogen-free (SPF) barrier facility at the Animal Facility of the Lung Cancer Research Institute, Tianjin Medical University General Hospital, under controlled conditions, including a temperature of 20 °C ± 2 °C, relative humidity of 40%–70%, and a 12 h light/dark cycle. Mice had free access to sterilized standard chow and autoclaved purified water. After 1 week of acclimatization, mice were randomly assigned to the designated experimental groups. All animal experiments were approved by the Animal Ethics and Welfare Committee of Tianjin Medical University General Hospital (Approval No. IRB2026-DW-54).

#### 2.2.1 LOP-induced STC-like mouse model and ACT treatment

After 1 week of acclimatization, male C57BL/6 mice were randomly divided into four groups: control (CON), STC, low-dose ACT treatment (ACTL), and high-dose ACT treatment (ACTH). During the modeling phase, mice in the STC, ACTL, and ACTH groups were administered LOP at 10 mg/kg once daily for 7 days to establish an STC-like model, as previously described with minor modifications, whereas CON mice received an equivalent volume of saline (Hjørne et al., 2025; Ren et al., 2022). After the 7-day modeling phase, mice entered a 7-day treatment phase. During this period, STC mice received LOP plus saline, ACTL and ACTH mice received LOP combined with ACT at 30 and 60 mg/kg, respectively, and CON mice continued to receive saline. The ACT doses were selected based on previous mouse studies reporting protective and anti-inflammatory effects of ACT at 30 and 60 mg/kg (Guo et al., 2022; Guo et al., 2025). At the experimental endpoint, mice were deeply anesthetized by intraperitoneal injection of sodium pentobarbital (50 mg/kg), and adequate anesthesia was confirmed by the absence of the pedal withdrawal reflex. Blood samples were collected from the retro-orbital plexus for serum preparation, after which the mice were euthanized by cervical dislocation under deep anesthesia. Death was confirmed before intestinal tissues were collected and stored at −80 °C for subsequent analyses.

#### 2.2.2 *Nlrp3*-KO mouse experiment for genetic validation

For genetic validation, wild-type (WT) and *Nlrp3*-KO mice were randomly assigned to four groups: WT-STC, WT-STC + ACTH, KO-STC, and KO-STC + ACTH. The same LOP-induced STC-like modeling protocol described above was applied. During the intervention period, WT-STC and KO-STC mice received saline, whereas WT-STC + ACTH and KO-STC + ACTH mice received ACT at 60 mg/kg. After fasting, defecation and gastrointestinal transit tests were performed. At the experimental endpoint, mice were anesthetized and euthanized using the same procedures described above, after which intestinal tissues were collected and stored at −80 °C for subsequent analyses.

### 2.3 General condition monitoring and fecal parameter measurement

Body weight (BW) was recorded at predetermined time points throughout the experiment to monitor the general condition of the mice (Frith et al., 2025). After the final administration, mice were fasted without food or water for 16 h. A fresh India ink suspension (0.4 mg/mL; Shanghai Yuanye Bio-Technology Co., Ltd., Shanghai, China) was prepared, and each mouse was orally gavaged with 0.2 mL of the suspension. The interval from gavage to the first appearance of black feces was recorded as the time to first dark stool. Fecal pellets were collected within 3 h after India ink administration, and the number and wet weight of fecal pellets were recorded immediately. The samples were then dried at 60 °C to a constant weight, and fecal water content was calculated as follows: Fecal water content (%) $=\frac{\text{Wet}\;\text{weight}\;\text{of}\;\text{feces}-\text{dry}\;\text{weight}\;\text{of}\;\text{feces}}{\text{Wet}\;\text{weight}\;\text{of}\;\text{feces}}$ × 100%

### 2.4 Intestinal propulsion test

After fecal sample collection, each mouse was orally administered an additional 0.2 mL of fresh India ink suspension. Twenty minutes later, the mice were anesthetized and euthanized using the same procedures described above. The intestinal segment from the pylorus to the cecum was carefully removed without stretching. The total intestinal length and the distance traveled by the India ink were measured, and the intestinal propulsion rate was calculated as follows: Intestinal propulsion rate (%) $=\frac{\text{India}\;\text{ink}\;\text{propulsion}\;\text{distance}}{\text{Total}\;\text{intestinal}\;\text{length}}$ ×100%

### 2.5 ELISA

At the experimental endpoint, blood samples were collected from the retro-orbital plexus into tubes without anticoagulant. After clotting at room temperature for 30 min, the samples were centrifuged at 3,000 rpm for 10 min at 4 °C. Serum was collected and immediately used for cytokine detection. Serum levels of IL-1β, IL-6, and TNF-α were measured using ELISA kits according to the manufacturer’s instructions. Optical density was measured at 450 nm using a microplate reader, and cytokine concentrations were calculated from standard curves. The intra- and inter-assay coefficients of variation were both less than 10%.

### 2.6 Hematoxylin and eosin (H&E) staining of colonic tissues

Colonic tissues were fixed in 4% paraformaldehyde for 48 h and processed for paraffin embedding. Tissue sections with a thickness of 5 μm were prepared and stained using a H&E staining kit (Cat# G1120, Solarbio) according to the manufacturer’s instructions. The stained sections were observed under a light microscope to evaluate colonic histopathological alterations, and representative images were captured for analysis.

### 2.7 Quantitative reverse transcription polymerase chain reaction (qRT-PCR) assay

Total RNA was isolated from mouse colonic tissues using a rapid tissue RNA extraction kit (Cat# AC0202, SparkJade, China) according to the manufacturer’s instructions. RNA concentration and purity were determined using a NanoDrop 2000 spectrophotometer (Thermo Fisher Scientific, United States). cDNA was synthesized using SPARKscript II All-in-one RT SuperMix for qPCR with gDNA Eraser (Cat# AG0305, SparkJade, China). Quantitative real-time PCR was performed using SYBR Green qPCR Mix (Cat# AH0104, SparkJade, China). *Gapdh* was used as the internal reference gene, and relative mRNA expression was calculated using the 2^−ΔΔCT^ method. Primer sequences were designed based on NCBI RefSeq reference transcripts and synthesized by Shanghai Sangon Biotech Co., Ltd. Detailed primer information is listed in Table 1.

**TABLE 1:** Primer sequences.

Gene	Primer	Sequence (5′–3′)
*Nlrp3*	Forward	TCA​CAA​CTC​GCC​CAA​GGA​GGA​A
	Reverse	AAG​AGA​CCA​CGG​CAG​AAG​CTA​G
*Il1b*	Forward	TGG​ACC​TTC​CAG​GAT​GAG​GAC​A
	Reverse	GTT​CAT​CTC​GGA​GCC​TGT​AGT​G
*Il6*	Forward	TAC​CAC​TTC​ACA​AGT​CGG​AGG​C
	Reverse	CTG​CAA​GTG​CAT​CAT​CGT​TGT​TC
*Tnf*	Forward	GGT​GCC​TAT​GTC​TCA​GCC​TCT​T
	Reverse	GCC​ATA​GAA​CTG​ATG​AGA​GGG​AG
*Gapdh*	Forward	CAT​CAC​TGC​CAC​CCA​GAA​GAC​TG
	Reverse	ATG​CCA​GTG​AGC​TTC​CCG​TTC​AG

### 2.8 Western blot detection

Colonic tissues were homogenized in RIPA lysis buffer (Cat# EA0002, SparkJade, China) supplemented with protease and phosphatase inhibitors. Protein concentrations were determined using a BCA protein assay kit (Cat# EC0001, SparkJade, China). Equal amounts of protein (30 μg per lane) were mixed with 5× SDS-PAGE loading buffer (Cat# EC0013, SparkJade, China) and boiled at 100 °C for 10 min. Proteins were separated by SDS-PAGE and transferred onto polyvinylidene difluoride (PVDF) membranes under ice-cooled conditions. After blocking with 5% non-fat milk for 1 h at room temperature, the membranes were incubated with primary antibodies overnight at 4 °C. After washing with Tris-buffered saline containing 0.1% Tween-20 (TBST), the membranes were incubated with an HRP-conjugated secondary antibody for 1 h at room temperature. Protein bands were visualized using an ECL substrate (Cat# ED0015, SparkJade, China) and detected with a Syngene G imaging system. β-actin was used as the internal loading control, and band intensities were quantified using ImageJ software.

### 2.9 Immunofluorescence staining

Fresh colonic tissues were rinsed with oxygenated phosphate-buffered saline (PBS) to remove luminal contents. The tissues were pinned onto a silicone-coated plate, and the longitudinal muscle–myenteric plexus (LMMP) layer was carefully dissected under a stereomicroscope. The isolated LMMP preparations were fixed in 4% paraformaldehyde overnight and washed three times with PBS. For immunofluorescence staining, LMMP tissues were permeabilized with 0.5% Triton X-100 for 1 h at room temperature and blocked with 5% bovine serum albumin (BSA) for 2 h. Separate LMMP preparations were incubated overnight at 4 °C with the corresponding primary antibodies against HuC/D or PGP9.5. After washing with PBS, the tissues were incubated with CoraLite 488-conjugated goat anti-rabbit IgG for 1 h at room temperature. Nuclei were counterstained using DAPI-containing mounting medium. Images were acquired using a fluorescence microscope, and fluorescence signals were quantified using ImageJ software.

### 2.10 Transcriptomic sequencing and bioinformatic analysis

Colonic tissues from the CON, STC, and ACTH groups were collected for RNA sequencing. Total RNA was extracted from colonic tissues, and RNA concentration, purity, and integrity were assessed before library construction. Qualified RNA samples were used for cDNA library preparation and sequenced on an Illumina platform. Raw reads were filtered to remove adaptor sequences and low-quality reads, and the resulting clean reads were aligned to the *Mus musculus* reference genome GRCm38 (mm10) with Ensembl release 100 annotations using STAR. Gene counts were generated using HTSeq, and gene expression levels were quantified as fragments per kilobase of transcript per million mapped reads (FPKM) for downstream analysis.

Differential expression analysis was performed using DESeq2. Genes with an FDR-adjusted q value <0.05 and |log_2_ fold change| > 1 were defined as differentially expressed genes. To identify disease-associated genes suppressed by ACTH treatment, genes upregulated in the STC vs. CON comparison were intersected with genes downregulated in the ACTH vs. STC comparison. The overlapping genes were defined as STC-upregulated and ACTH-suppressed genes and visualized by hierarchical clustering. Protein-protein interaction (PPI) analysis of these genes was performed using the STRING database, with *M. musculus* as the reference organism and a minimum interaction score of 0.15. Gene Ontology (GO) enrichment analysis was performed to identify enriched biological processes. Gene set enrichment analysis (GSEA) was conducted using ranked gene lists to evaluate pathway-level alterations, with particular attention to inflammation-related pathways, including the NOD-like receptor signaling pathway. Exploratory weighted gene co-expression network analysis was performed using the WGCNA package in R, with a soft-thresholding power of 9, a minimum module size of 60, and a module merge cut height of 0.25. Module eigengenes were correlated with the experimental groups, and the blue and green modules showing the strongest associations with ACTH treatment were subjected to GO biological process and KEGG pathway enrichment analyses. Volcano plots, hierarchical clustering heatmaps, GO enrichment plots, GSEA plots, PPI networks, and WGCNA-related plots were generated to visualize the transcriptomic results. RNA sequencing and preliminary data processing were performed by Shanghai Liebing Biomedical Technology Co., Ltd.

### 2.11 Statistical analysis

All data are presented as the mean ± standard deviation (SD). Statistical analyses were performed using GraphPad Prism 8.0 (GraphPad Software, United States). For comparisons among the CON, STC, ACTL, and ACTH groups, one-way analysis of variance (ANOVA) followed by Tukey’s multiple-comparison test was used. For *Nlrp3*-KO experiments, two-way ANOVA followed by Tukey’s multiple-comparison test was performed, with genotype and ACTH treatment as the two factors. A value of P < 0.05 was considered statistically significant.

## 3 Results

### 3.1 ACT alleviates LOP-induced STC-like phenotypes in mice

**FIGURE 1:**
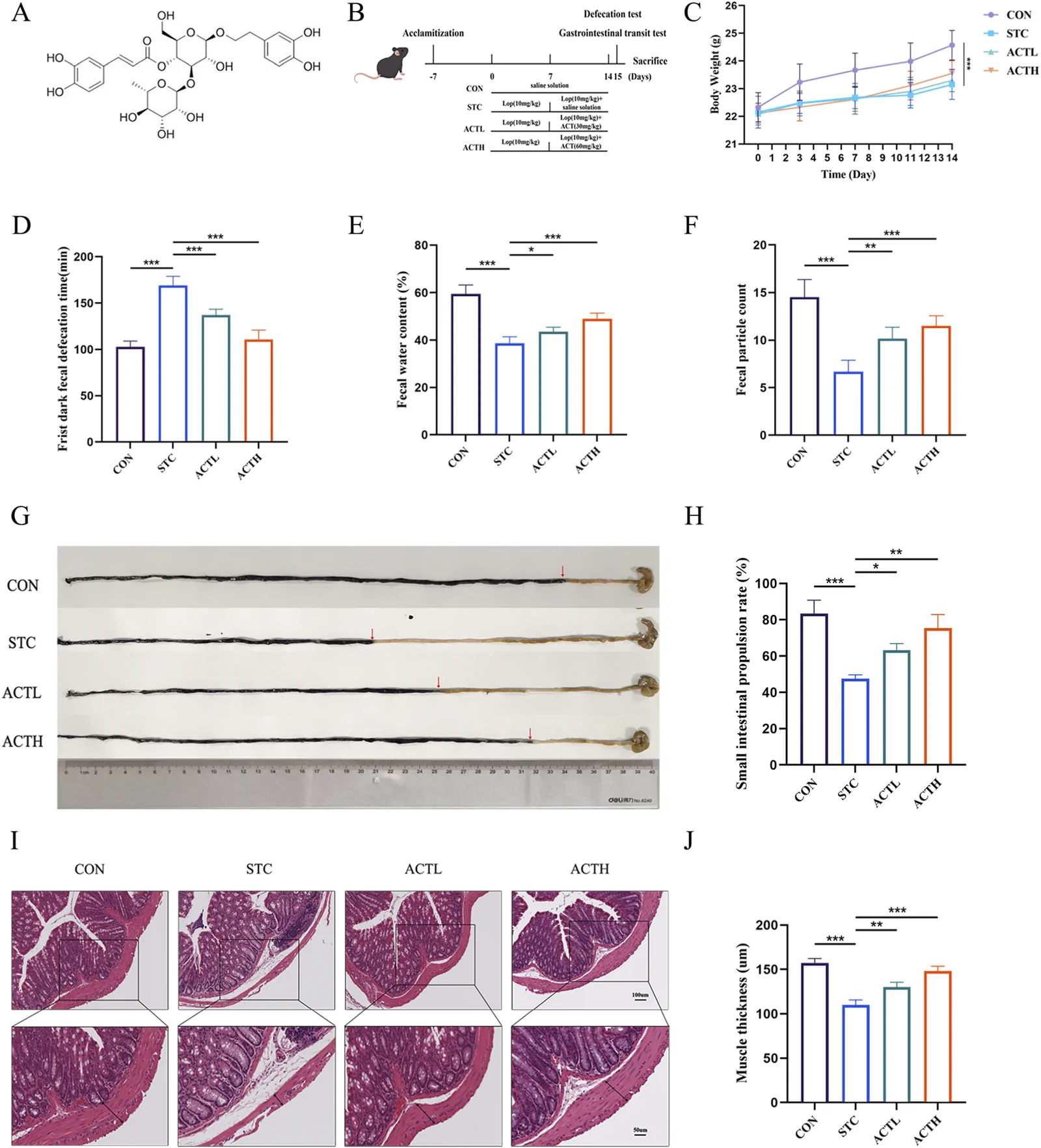
ACT alleviates LOP-induced STC-like phenotypes in mice. **(A)** Molecular structure of ACT; **(B)** Flow chart of the experimental design; **(C)** BW changes during the experimental period (n = 6); **(D)** First dark fecal defecation time (min) (n = 6); **(E)** Fecal water content (%) (n = 6); **(F)** Fecal particle count (n = 6); **(G)** Representation of India ink in the intestines after gavage, red arrows indicate the location where India ink reached (n = 3); **(H)** Small intestinal propulsion rate (%) (n = 3); **(I)** Representative H&E-stained images of colonic tissues (n = 3); and **(J)** Quantification of colonic muscular layer thickness (μm) (n = 3). Data are presented as the mean ± SD. Statistical significance was determined at P < 0.05. *P < 0.05, **P < 0.01, ***P < 0.001. CON, control group; STC, LOP-induced STC-like model group; ACTL, low-dose ACT treatment group; ACTH, high-dose ACT treatment group.

The chemical structure of ACT and the experimental design are shown in Figures 1A,B. During the experimental period, no obvious differences in BW were observed among the groups, suggesting that ACT did not exert an apparent adverse effect on BW (Figure 1C). Compared with the CON group, LOP-treated STC mice exhibited typical constipation-like phenotypes, including a prolonged time to first dark stool, reduced fecal water content, decreased fecal pellet number, and impaired small-intestinal propulsion (Figures 1D–H). ACT administration significantly improved these abnormalities, with the high-dose ACT group showing a more pronounced effect.

H&E staining was further performed to evaluate colonic histopathological changes. The CON group displayed an intact colonic structure, characterized by a continuous muscular layer, well-organized crypt architecture, and orderly arranged epithelial cells (Figure 1I). In contrast, LOP-treated STC mice showed apparent histological abnormalities, including thinning of the muscular layer, disrupted crypt architecture, epithelial disorganization, and mild inflammatory cell infiltration. ACT treatment alleviated these pathological alterations, as reflected by improved mucosal architecture and partial restoration of colonic muscular layer thickness, particularly in the ACTH group (Figures 1I,J). Together, these results indicate that ACT alleviated LOP-induced STC-like phenotypes and improved intestinal transit function, with the high-dose treatment showing greater efficacy.

### 3.2 ACT improves colonic myenteric neuropathy in LOP-induced STC-like mice

**FIGURE 2:**
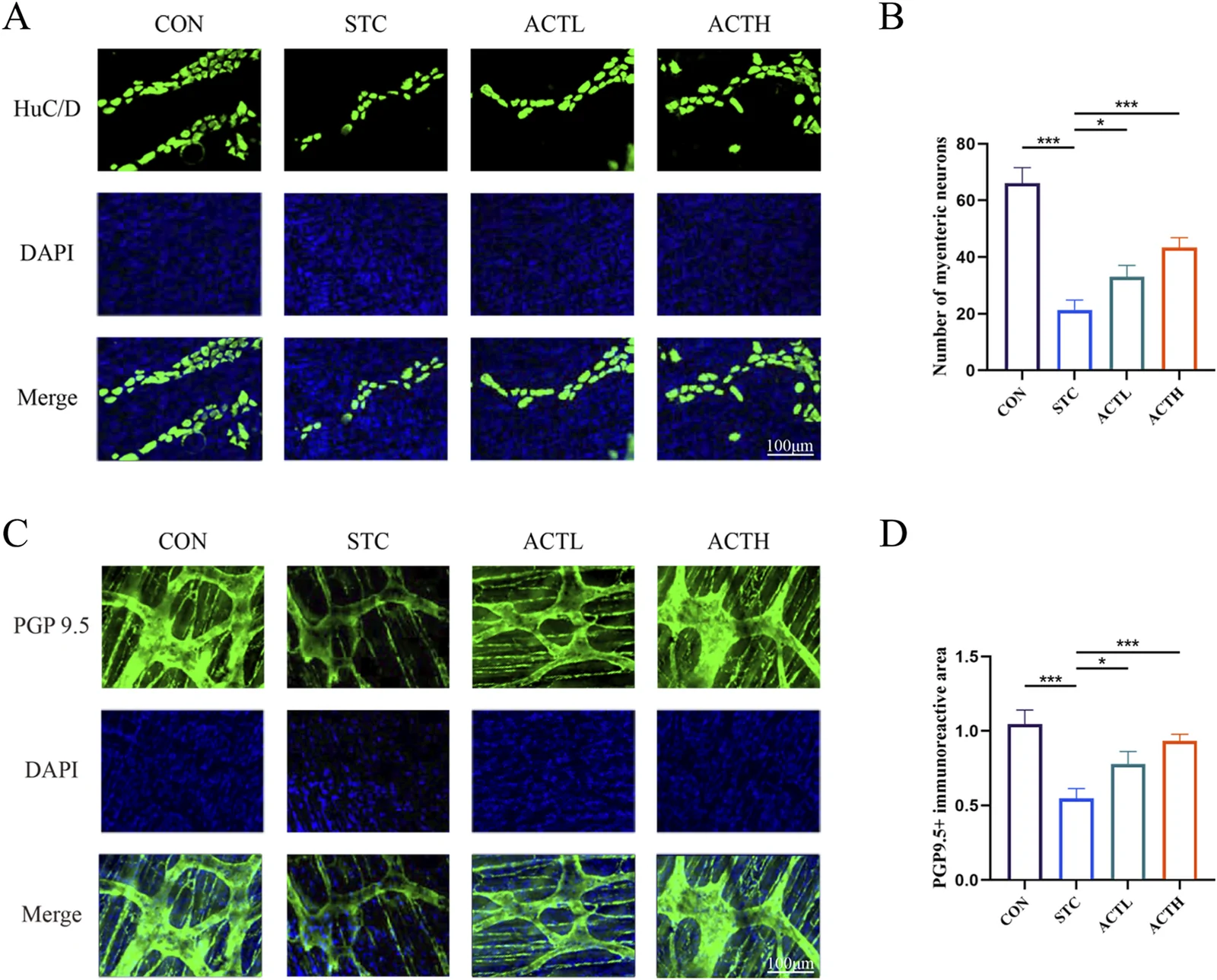
ACT improves colonic myenteric neuropathy in LOP-induced STC-like mice. **(A)** LMMP preparations followed by immunofluorescence staining of myenteric neurons with HuC/D (green) and DAPI (blue) as representative images (Scale bars: 100 μm) (n = 3); **(B)** Quantification of HuC/D-positive myenteric neurons (n = 3); **(C)** LMMP preparations followed by immunofluorescence staining of enteric nerve fibers with PGP9.5 (green) and DAPI (blue) as representative images (Scale bars: 100 μm) (n = 3); and **(D)** Quantification of PGP9.5-positive immunoreactive area (n = 3). Data are expressed as mean ± SD. Statistical significance was determined at P < 0.05. *P < 0.05, **P < 0.01, ***P < 0.001. CON, control group; STC, LOP-induced STC-like model group; ACTL, low-dose ACT treatment group; ACTH, high-dose ACT treatment group.

To determine whether ACT improved constipation-like phenotypes by preserving the enteric nervous system, immunofluorescence staining for HuC/D and PGP9.5 was performed. Compared with the CON group, LOP-treated STC mice showed a marked reduction in HuC/D-positive myenteric neurons, indicating enteric neuronal loss or injury (Figures 2A,B). ACT treatment significantly increased the number of HuC/D-positive neurons, with a more pronounced effect observed in the ACTH group.

Consistently, PGP9.5 staining showed that the enteric nerve fiber network was markedly weakened in STC mice, as reflected by a reduced PGP9.5-positive immunoreactive area (Figures 2C,D). ACT administration partially reversed this reduction, especially at the high dose. These results suggest that ACT treatment is associated with the preservation of myenteric neurons and enteric nerve fibers in LOP-induced STC-like mice.

### 3.3 Transcriptomic analysis identifies NLRP3-related inflammatory signaling and broader ACTH-responsive networks

**FIGURE 3:**
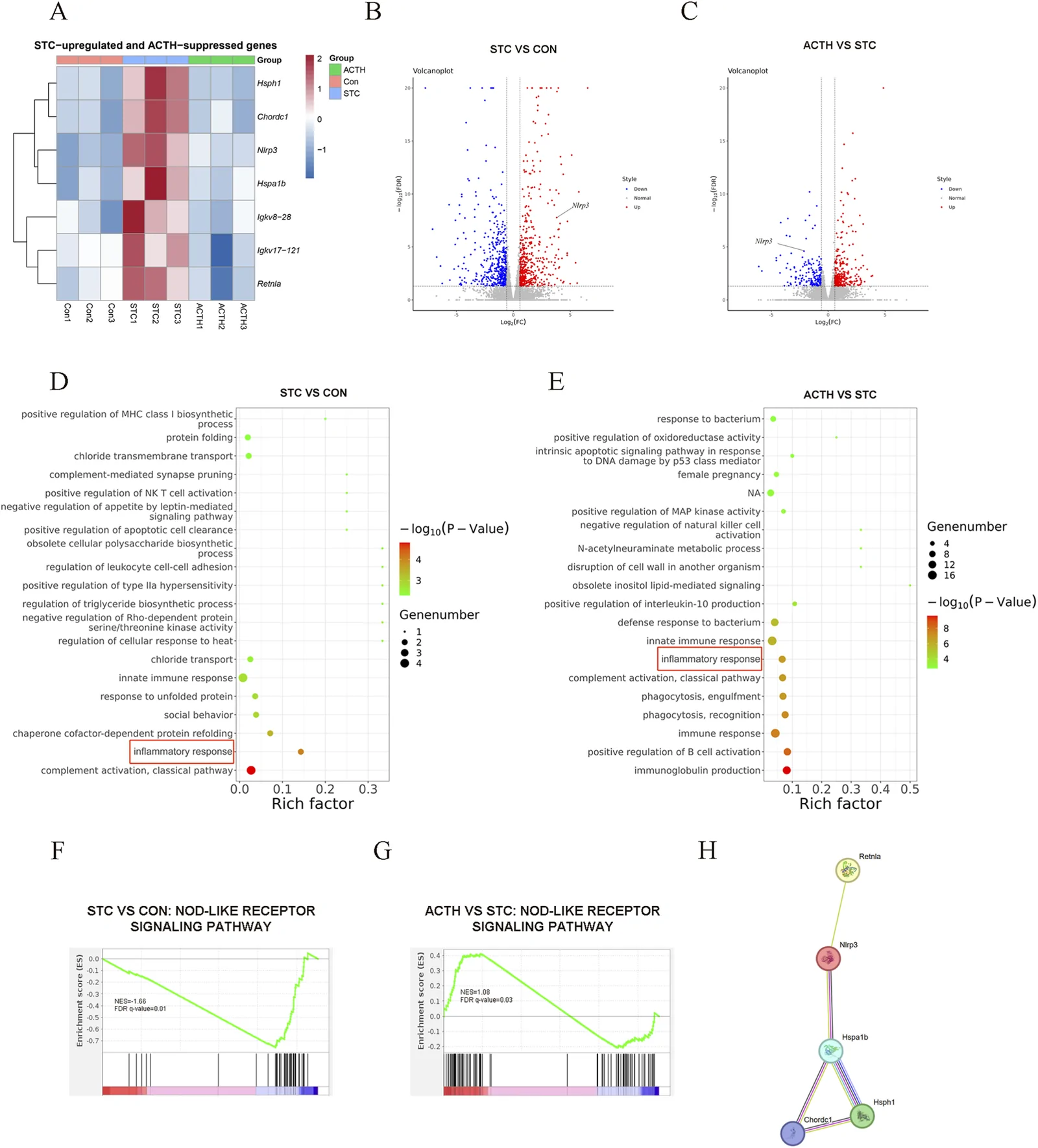
Transcriptomic analysis identifies NLRP3-related inflammatory signaling and associated molecular networks. **(A)** Hierarchical clustering heatmap of genes upregulated in the STC vs. CON comparison and downregulated in the ACTH vs. STC comparison; **(B,C)** Volcano plots of differentially expressed genes in STC vs. CON and ACTH vs. STC comparisons; **(D,E)** GO enrichment analyses of differentially expressed genes in STC vs. CON and ACTH vs. STC comparisons; **(F,G)** GSEA of the NOD-like receptor signaling pathway in STC vs. CON and ACTH vs. STC comparisons; **(H)** STRING-based predicted PPI network of the connected overlapping genes. *Igkv8-28* and *Igkv17-121* were not displayed because no predicted interactions were identified. CON: control group; STC: LOP-induced STC-like model group; ACTH: high-dose ACT treatment group.

Because ACTH showed more pronounced protective effects, colonic transcriptomic analysis was performed in the CON, STC, and ACTH groups. Genes upregulated in the STC vs. CON comparison were intersected with genes downregulated in the ACTH vs. STC comparison. Seven overlapping genes were identified, including *Hsph1, Chordc1, Nlrp3, Hspa1b, Igkv8-28, Igkv17-121*, and *Retnla*. Hierarchical clustering confirmed their elevation in the STC group and reduction following ACTH treatment (Figure 3A). Volcano plots further showed that *Nlrp3* was upregulated in STC mice and downregulated after ACTH treatment (Figures 3B,C).

GO enrichment analysis identified immune- and inflammation-related biological processes in both comparisons (Figures 3D,E). GSEA further indicated significant alterations in the NOD-like receptor signaling pathway in the STC vs. CON and ACTH vs. STC comparisons (Figures 3F,G).

STRING-based PPI analysis showed that five of the seven overlapping genes formed a predicted network involving *Nlrp3, Retnla, Hspa1b, Hsph1*, and *Chordc1* (Figure 3H). Exploratory WGCNA independently identified a blue module positively associated with ACTH treatment (r = 0.67, P = 0.05) and a green module showing an inverse association trend (r = −0.65, P = 0.06) (Supplementary Figures S1A,B). The blue module was enriched in immune, inflammatory, intestinal absorptive, metabolic, and serotonergic processes, whereas the green module was mainly associated with immune-related processes (Supplementary Figures S1C-E). Collectively, these findings identify NLRP3-related inflammatory signaling as a prominent ACTH-responsive alteration within broader transcriptional networks.

### 3.4 ACTH suppresses NLRP3 inflammasome activation and inflammatory cytokine production

**FIGURE 4:**
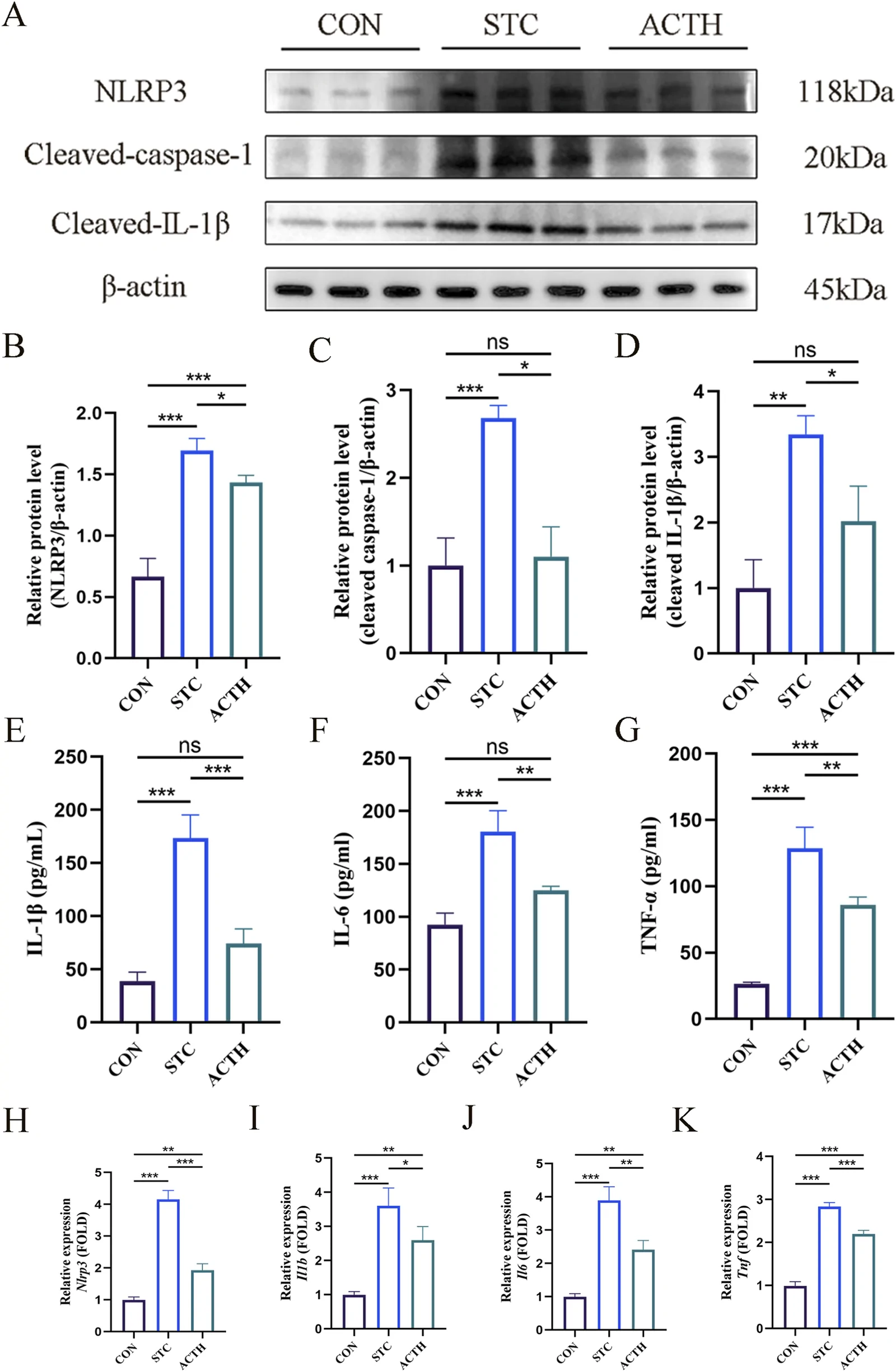
ACTH suppresses NLRP3 inflammasome activation and inflammatory cytokine production. **(A)** Representative Western blot images of NLRP3, cleaved-caspase-1, cleaved-IL-1β, and β-actin (n = 3); **(B–D)** Quantification of NLRP3, cleaved-caspase-1, and cleaved-IL-1β protein levels (n = 3); **(E–G)** Serum levels of IL-1β, IL-6, and TNF-α detected by ELISA (n = 3); and **(H–K)** Relative mRNA expression levels of *Nlrp3, Il1b, Il6, and Tnf* detected by qRT-PCR (n = 3). Data are expressed as mean ± SD. Statistical significance was determined at P < 0.05. *P < 0.05, **P < 0.01, ***P < 0.001; ns, not significant. CON, control group; STC, LOP-induced STC-like model group; ACTH, high-dose ACT treatment group.

Given that *Nlrp3* was among the STC-upregulated and ACTH-suppressed genes and that the NOD-like receptor signaling pathway was altered, we next examined whether ACTH affected NLRP3 inflammasome activation in LOP-induced STC-like mice. Western blot analysis showed that the protein levels of NLRP3, cleaved caspase-1, and cleaved IL-1β were markedly increased in the STC group compared with the CON group, indicating activation of the NLRP3 inflammasome pathway. ACTH treatment significantly reduced the levels of these inflammasome-related proteins (Figures 4A–D).

Consistently, ELISA results showed that serum levels of IL-1β, IL-6, and TNF-α were elevated in STC mice, whereas ACTH administration significantly decreased these inflammatory cytokines (Figures 4E–G). qRT-PCR analysis further confirmed that the mRNA expression levels of *Nlrp3, Il1b, Il6, and Tnf* were upregulated after LOP treatment and were partially reversed by ACTH (Figures 4H–K). These results indicate that ACTH attenuates NLRP3 inflammasome activation and suppresses inflammatory responses in LOP-induced STC-like mice.

### 3.5 *Nlrp3* deficiency partially recapitulates ACTH-mediated improvement of LOP-induced STC-like phenotypes

**FIGURE 5:**
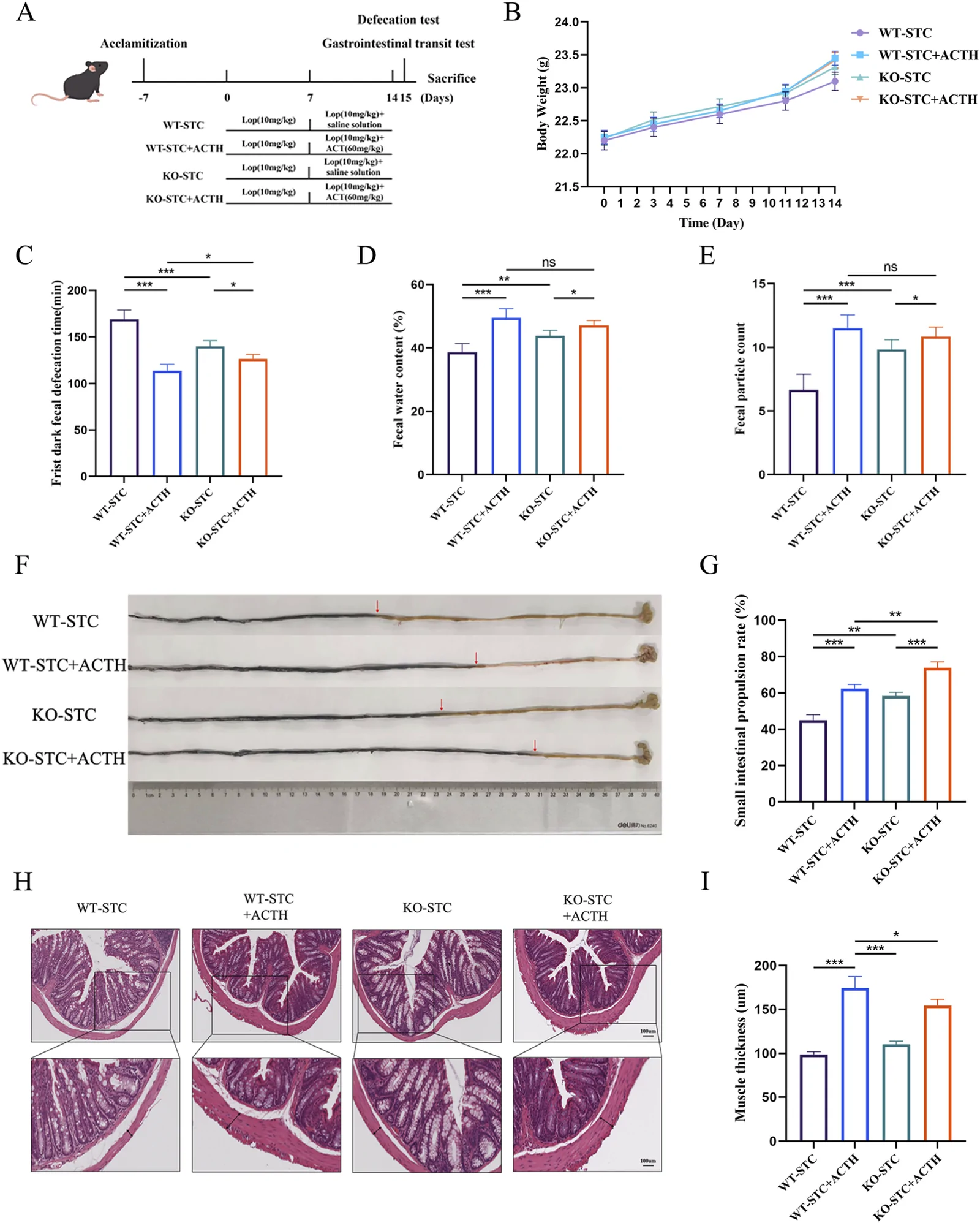
*Nlrp3* deficiency partially recapitulates ACTH-mediated improvement of LOP-induced STC-like phenotypes. **(A)** Flow chart of the experimental design; **(B)** BW changes during the experimental period (n = 6); **(C)** First dark fecal defecation time (min) (n = 6); **(D)** Fecal water content (%) (n = 6); **(E)** Fecal particle count (n = 6); **(F)** Representation of India ink in the intestines after gavage, red arrows indicate the location where India ink reached (n = 3); **(G)** Small intestinal propulsion rate (%) (n = 3); **(H)** Representative H&E-stained images of colonic tissues (n = 3); and **(I)** Quantification of colonic muscular layer thickness (μm) (n = 3). Data are presented as the mean ± SD. Statistical significance was determined at P < 0.05. *P < 0.05, **P < 0.01, ***P < 0.001; ns, not significant. WT-STC, wild-type STC-like model group; WT-STC + ACTH, wild-type STC-like model group treated with high-dose ACT; KO-STC, *Nlrp3*-KO STC-like model group; KO-STC + ACTH, *Nlrp3*-KO STC-like model group treated with high-dose ACT.

To further determine whether NLRP3 contributes to the protective effects of ACTH, *Nlrp3*-deficient mice were used for genetic validation (Figure 5A). No significant group differences in BW were observed among WT-STC, WT-STC + ACTH, KO-STC, and KO-STC + ACTH mice during the experimental period (Figure 5B). Compared with WT-STC mice, ACTH-treated WT mice showed a shortened time to first dark stool, increased fecal water content and fecal pellet number, and improved small-intestinal propulsion (Figures 5C–G).

Under LOP-induced STC-like conditions, *Nlrp3* deficiency also attenuated constipation-like phenotypes, as evidenced by reduced defecation latency, increased fecal water content and fecal pellet number, and enhanced intestinal propulsion in KO-STC mice compared with WT-STC mice (Figures 5C–G). ACTH treatment further improved several parameters in *Nlrp3*-deficient mice, indicating that ACTH retained partial protective activity even in the absence of NLRP3. Histopathological examination further supported these functional findings. H&E staining showed that WT-STC mice exhibited colonic structural alterations, including a relatively thin muscular layer and disorganized mucosal architecture, whereas ACTH treatment partially restored colonic morphology in WT-STC mice (Figure 5H). In *Nlrp3*-deficient mice, ACTH treatment also improved the colonic histological appearance, with increased muscular layer thickness compared with KO-STC mice (Figures 5H,I). These findings suggest that *Nlrp3* deficiency partially reproduces the beneficial effects of ACTH and supports the involvement of NLRP3 signaling in ACTH-mediated improvement of LOP-induced STC-like phenotypes.

### 3.6 *Nlrp3* deficiency modifies ACTH-mediated inflammasome suppression and myenteric neuroprotection

**FIGURE 6:**
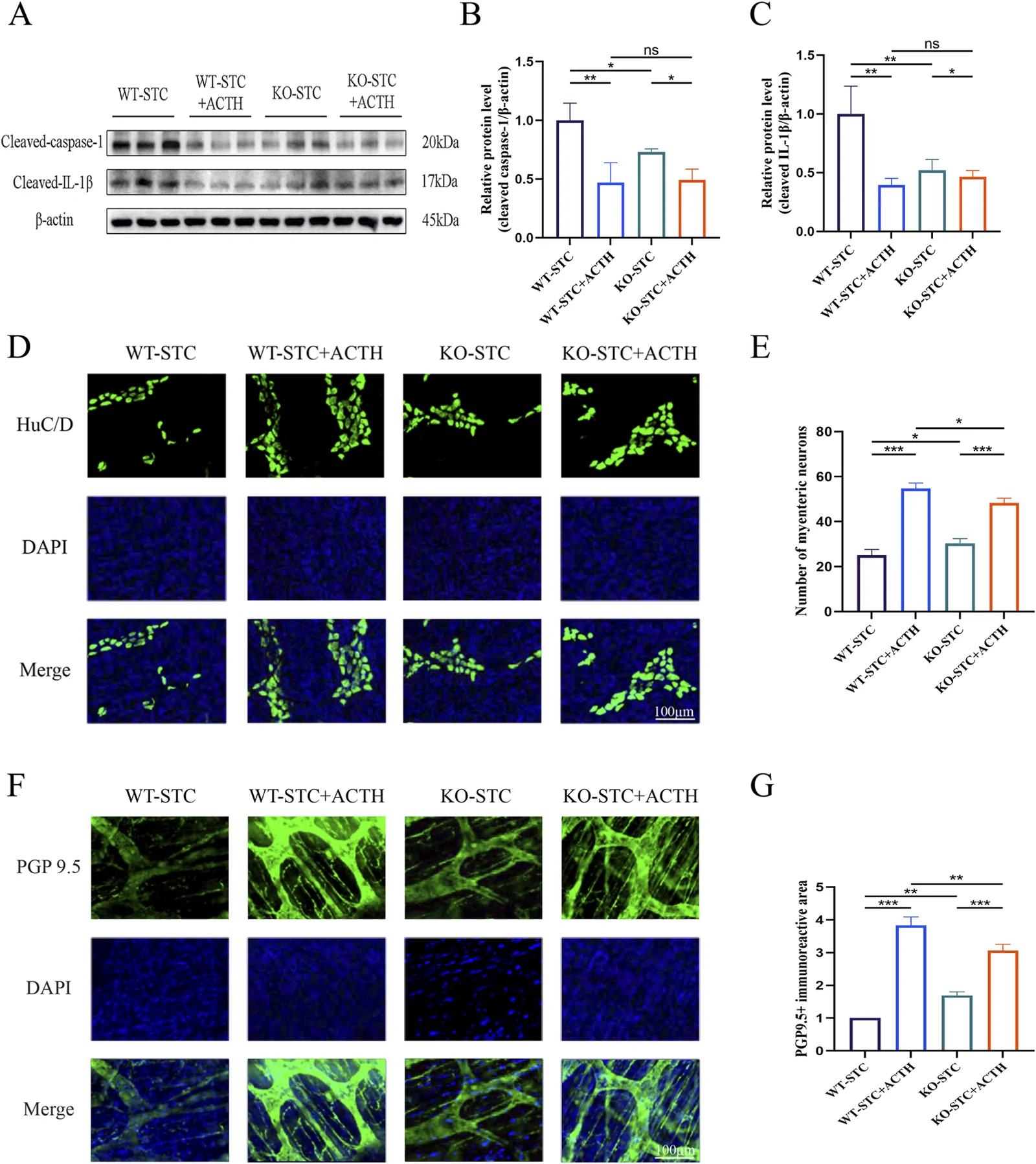
*Nlrp3* deficiency modifies ACTH-mediated inflammasome suppression and myenteric neuroprotection. **(A)** Representative Western blot images of cleaved caspase-1, cleaved-IL-1β, and β-actin (n = 3); **(B,C)** Quantification of cleaved-caspase-1 and cleaved-IL-1β protein levels (n = 3); **(D)** LMMP preparations followed by immunofluorescence staining of myenteric neurons with HuC/D (green) and DAPI (blue) as representative images (Scale bars: 100 μm) (n = 3); **(E)** Quantification of HuC/D-positive myenteric neurons (n = 3); **(F)** LMMP preparations followed by immunofluorescence staining of enteric nerve fibers with PGP9.5 (green) and DAPI (blue) as representative images (Scale bars: 100 μm) (n = 3); and **(G)** Quantification of PGP9.5-positive immunoreactive area (n = 3). Data are presented as the mean ± SD. Statistical significance was determined at P < 0.05. *P < 0.05, **P < 0.01, ***P < 0.001; ns, not significant. WT-STC, wild-type STC-like model group; WT-STC + ACTH, wild-type STC-like model group treated with high-dose ACT; KO-STC, *Nlrp3*-KO STC-like model group; KO-STC + ACTH, *Nlrp3*-KO STC-like model group treated with high-dose ACT.

To further evaluate the involvement of NLRP3 in ACTH-mediated ENS protection, inflammasome-related proteins and enteric neuronal markers were examined in WT-STC, WT-STC + ACTH, KO-STC, and KO-STC + ACTH mice. Western blot analysis showed that ACTH reduced the levels of cleaved caspase-1 and cleaved IL-1β in WT-STC mice (Figures 6A–C). Compared with WT-STC mice, KO-STC mice also exhibited lower levels of these proteins, indicating attenuation of inflammasome activation after *Nlrp3* deficiency. ACTH further reduced the residual levels of cleaved caspase-1 and cleaved IL-1β in KO-STC mice (Figures 6A–C).

HuC/D staining showed that ACTH increased the number of HuC/D-positive myenteric neurons in WT-STC mice (Figures 6D,E). KO-STC mice showed partial preservation of HuC/D-positive neurons compared with WT-STC mice, and ACTH further increased neuronal counts in KO-STC mice. Similarly, PGP9.5 staining showed that ACTH increased the PGP9.5-positive enteric nerve fiber area in both WT-STC and KO-STC mice (Figures 6F,G). Together, these results suggest that *Nlrp3* deficiency partially attenuates inflammasome activation and ENS injury, while ACTH exerts additional protective effects even in *Nlrp3*-KO mice.

## 4 Discussion

**FIGURE 7:**
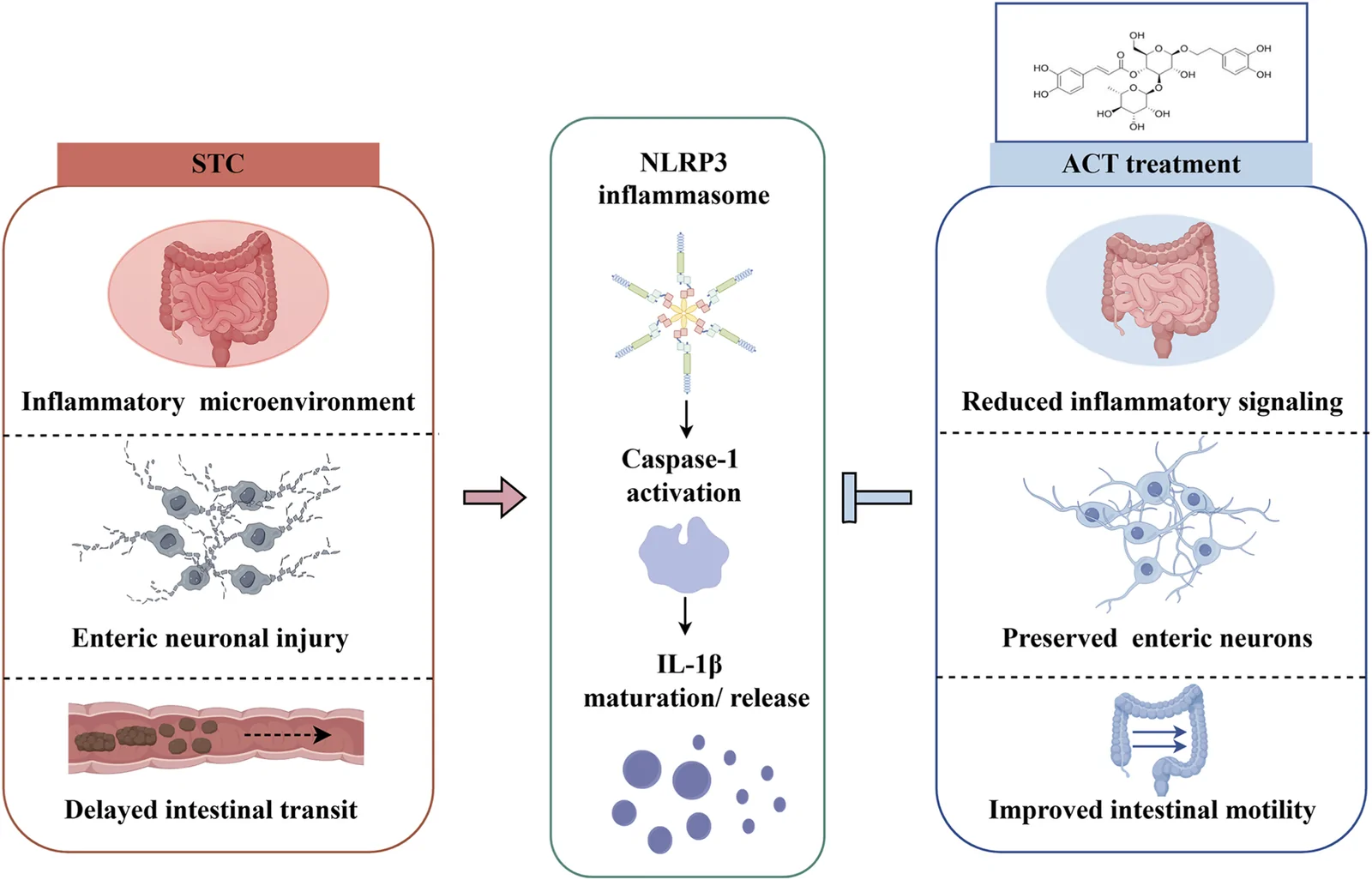
Schematic diagram summarizing the proposed association among ACT-mediated protection, NLRP3-associated inflammasome attenuation, ENS preservation, and potential NLRP3-independent mechanisms in STC-like injury.

STC-like intestinal dysmotility should be interpreted as a coordinated pathological process involving impaired propulsion, inflammatory remodeling, and enteric neuromuscular injury. Previous studies using LOP-induced constipation models have shown that constipation-like phenotypes are often accompanied by inflammatory activation, epithelial barrier disruption, altered water-electrolyte transport, gut microbiota changes, and neuroendocrine dysregulation (Choi et al., 2022; Li et al., 2022; Dong et al., 2023; Makizaki et al., 2021). However, relatively few studies have integrated inflammatory pathway screening with direct assessment of ENS integrity and genetic validation of a defined inflammatory target. In the present study, ACT improved defecation parameters and intestinal propulsion, attenuated colonic histological injury, preserved HuC/D-positive myenteric neurons and PGP9.5-positive enteric nerve fibers, and suppressed the NLRP3–caspase-1–IL-1β axis. Together, these findings suggest that ACT-mediated protection against LOP-induced STC-like injury is associated with coordinated attenuation of inflammatory signaling and preservation of enteric neuronal structures (Figure 7).

The improvement in intestinal transit induced by ACT is consistent with, but mechanistically distinct from, several previously reported anti-constipation interventions. For example, a water extract of Cannabis sativa L. improved LOP-induced constipation by regulating intestinal motility, water-electrolyte metabolism, inflammatory responses, and barrier-related alterations (Li et al., 2022). A lychee-derived phenolic complex was reported to alleviate LOP-induced constipation by reshaping the gut microbiome, increasing short-chain fatty acid production, and regulating ENS-related signaling and aquaporin expression (Dong et al., 2023). In addition, Atractylodes macrocephala insoluble dietary fiber was recently reported to ameliorate LOP-induced functional constipation in rats through mechanisms involving gut microbiota and bile acid metabolism (Wang et al., 2026). BBG9-1 also alleviated LOP-induced slow transit constipation in mice by reshaping the gut microbiota, increasing organic acid production, and improving neurotransmission-related alterations (Makizaki et al., 2021). Compared with these studies, the present work highlights a distinct but complementary mechanism: ACT-mediated restoration of intestinal transit was accompanied by suppression of inflammasome activation and preservation of myenteric neuronal structure. Therefore, ACT does not appear to act merely as a stool-promoting agent but may improve intestinal motility by targeting inflammation-associated neuromuscular injury.

The ENS findings are particularly important for interpreting the functional benefits of ACT. The ENS is the intrinsic neural network that coordinates gastrointestinal motility and local neuroimmune regulation (Sharkey and Mawe, 2023; Wang et al., 2022). Pathological studies have reported abnormalities in enteric nerves, myenteric plexuses, and interstitial cells of Cajal in patients with STC, supporting the concept that delayed transit is closely associated with enteric neuromuscular remodeling (He et al., 2000; Wedel et al., 2002). In experimental constipation models, ENS-targeted interventions have also been linked to motility restoration; for instance, sacral nerve stimulation improved LOP-induced constipation in rats, accompanied by ENS-related changes (Wang et al., 2023). In the present study, LOP-treated mice showed reduced numbers of HuC/D-positive myenteric neurons and weakened PGP9.5-positive enteric nerve fiber signals, whereas ACT partially restored both markers. These results provide structural evidence that ACT-mediated improvement in intestinal motility is accompanied by preservation of the myenteric plexus, consistent with the established involvement of ENS abnormalities in STC-related dysmotility.

Inflammatory signaling provides a plausible link between LOP-induced dysmotility and ENS impairment. LOP-induced constipation has been reported to activate colonic inflammatory signaling, including complement C3-related pathways, MAPK signaling, and increased inflammatory cytokine expression (Choi et al., 2022). In addition, inflammatory cytokines can directly modulate enteric neuronal function. IL-1β and IL-6 have been shown to excite enteric neurons while suppressing nicotinic and noradrenergic neurotransmission, and cytokine-driven immune activation has been broadly implicated in gastrointestinal motor dysfunction (Xia et al., 1999; Akiho et al., 2011; Tjwa et al., 2003). In the present study, ACTH reduced the levels of IL-1β, IL-6, and TNF-α while simultaneously preserving HuC/D and PGP9.5 signals. This coordinated pattern suggests that ACTH may protect the ENS by reducing inflammatory stress within the intestinal microenvironment, thereby supporting the recovery of intestinal propulsion.

Among the inflammatory pathways identified in this study, NLRP3 inflammasome signaling appears to represent a key molecular node. Thus, our data support the involvement of NLRP3 inflammasome attenuation in ACT-mediated protection, while additional upstream and downstream regulatory events remain to be investigated. NLRP3 promotes caspase-1 activation and the maturation of IL-1β and IL-18 and has been widely implicated in inflammatory tissue injury and gastrointestinal inflammation (Xu and Núñez, 2023; Li et al., 2025; Zhen and Zhang, 2019). Notably, a recent STC-related study reported that Batatasin III alleviated LOP-induced STC by inhibiting the NLRP3–IL-1β inflammatory pathway, restoring neurotransmitter levels, and improving enteric neuropathy (Yin et al., 2026). Our findings are consistent with this NLRP3-related inflammatory mechanism but provide two additional layers of evidence. First, further transcriptomic analysis identified *Nlrp3* among the STC-upregulated and ACTH-suppressed genes and placed it within a predicted PPI network involving inflammation- and cellular stress-related genes. Exploratory WGCNA independently identified broader ACTH-associated immune, metabolic, intestinal absorptive, and serotonergic transcriptional programs. These findings suggest that NLRP3-related signaling represents an important component of broader ACTH-responsive transcriptional alterations. Second, *Nlrp3*-KO mice were used to genetically validate the contribution of this pathway. Thus, the present study further strengthens the link between NLRP3 inflammasome activation, ENS injury, and STC-like motility dysfunction.

The *Nlrp3*-KO experiment further supports a partial-dependence model for ACT-mediated protection. This interpretation is consistent with recent evidence showing that *Nlrp3* deficiency can enhance gut motility and increase excitatory enteric neurons in association with preserved muscularis macrophages (Gao et al., 2024), and that NLRP3 signaling participates in immune and inflammatory responses and in enteric neuroplastic remodeling associated with colonic dysmotility (Pellegrini et al., 2021). In the present study, *Nlrp3* deficiency improved STC-like phenotypes, reduced the levels of cleaved caspase-1 and cleaved IL-1β, and partially preserved myenteric neurons and enteric nerve fibers under LOP-induced conditions. These results indicate that NLRP3 activation is not merely a secondary inflammatory marker but functionally contributes to intestinal dysmotility and ENS injury in this model. However, ACTH still exerted additional protective effects in *Nlrp3*-KO mice, including further reductions in residual cleaved caspase-1 and cleaved IL-1β levels. This pattern suggests that NLRP3 inflammasome suppression is an important, but not exclusive, mechanism underlying ACT-mediated protection, and that ACT may also modulate NLRP3-independent inflammatory, oxidative stress-related, or neuroimmune remodeling pathways.

This interpretation is consistent with related studies of ACT in inflammatory and neuroinflammatory injury models. In DSS-induced colitis, ACT ameliorated colonic inflammation by regulating the HO-1/HMGB1 pathway, supporting its ability to modulate intestinal inflammatory injury (Guo et al., 2022). In ischemic stroke, ACT protected against blood–brain barrier disruption by inhibiting microglial HMGB1/TLR4/NLRP3 signaling and reducing inflammatory and pyroptotic responses (Liao et al., 2024). Similarly, verbascoside attenuated acute inflammatory injury after intracerebral hemorrhage by suppressing NLRP3 activation (Zhou et al., 2021). These studies indicate that ACT can regulate inflammatory injury in both intestinal and neural contexts. The present study extends this concept to the enteric nervous system, showing that ACT treatment is associated with preservation of myenteric neurons and enteric nerve fibers within a constipation-associated inflammatory microenvironment.

Several limitations should be acknowledged. First, the LOP-induced model mainly reflects opioid receptor-mediated intestinal hypomotility and may not fully recapitulate the chronic neuromuscular remodeling and clinical heterogeneity of human STC. In addition, broader dose-ranging studies, together with pharmacokinetic and pharmacodynamic evaluations, are needed to further support the translational development of ACT. Second, although ENS abnormalities are closely associated with STC-related dysmotility, whether ENS preservation is required for ACT-mediated motility improvement remains to be clarified using neuron-specific ablation or rescue strategies. Third, although this study showed that ACT attenuated activation of the NLRP3/caspase-1/IL-1β axis, Western blotting and qRT-PCR analyses were performed with a limited number of biological replicates, and larger independent cohorts would help further validate the robustness of these molecular changes. In addition, the upstream events driving NLRP3 activation and downstream IL-18, NF-κB, or MAPK-related responses were not examined. Gut microbiota composition and intestinal barrier integrity were also not assessed; therefore, whether microbiota–barrier–neuroimmune interactions contribute to ACT-mediated protection remains to be further clarified. Fourth, systemic *Nlrp3*-KO mice were used in this study; therefore, the specific cellular source of NLRP3 signaling within the intestinal neuroimmune microenvironment remains to be clarified. In particular, whether ACT primarily modulates NLRP3 signaling in mucosal epithelial cells, muscularis macrophages, enteric glia, or myenteric neurons, and whether neuronal protection occurs through direct neuronal effects or indirect immune-mediated mechanisms, remains unclear. Future studies using cell-specific genetic approaches, primary enteric neuron-based systems, or neuroimmune co-culture models would help further define the cellular mechanisms underlying ACT-mediated enteric neuroprotection.

## 5 Conclusion

In conclusion, ACT alleviated LOP-induced STC-like phenotypes and improved intestinal transit in mice. At the molecular level, ACT attenuated NLRP3 inflammasome activation, reduced inflammatory cytokine production, and was associated with preservation of myenteric neurons and enteric nerve fibers. *Nlrp3* deficiency partially reproduced the protective effects of high-dose ACT, supporting the involvement of NLRP3 signaling in ACT-mediated enteric neuroprotection. These findings suggest that ACT ameliorates STC-like injury in association with attenuation of NLRP3 inflammasome activation and preservation of ENS integrity, while additional NLRP3-independent mechanisms may also contribute to its protective effects.

## Data Availability

The original contributions presented in the study are publicly available. This data can be found here: NCBI Sequence Read Archive (SRA), BioProject accession PRJNA1519138.

## Supplementary material

SUPPLEMENTARY FIGURE S1 Exploratory WGCNA and functional enrichment of ACTH-associated co-expression modules. **(A)** Gene dendrogram and merged module colors. **(B)** Module–trait relationships. Values represent correlation coefficients, with P values shown in parentheses. **(C)** GO biological process enrichment of the blue module. **(D)** KEGG pathway enrichment of the blue module. **(E)** GO biological process enrichment of the green module.
